# Differential effects of right atrial ablation sites on vagal modulation of the sinus node during cardioneuroablation

**DOI:** 10.1007/s10840-026-02341-7

**Published:** 2026-04-30

**Authors:** Peter Štiavnický, Dan Wichterle, Helena Jansová, Petr Peichl, Robert Čihák, Predrag Stojadinović, Josef Kautzner

**Affiliations:** 1 Department of Cardiology, Institute for Clinical and Experimental Medicine (IKEM), Vídeňská 1958/9, 140 21 Prague, Czechia; 2 Institute of Physiology, First Faculty of Medicine, Charles University, Prague, Czechia; 3 2nd Department of Medicine – Department of Cardiovascular Medicine, First Faculty of Medicine, Charles University, Prague, Czechia

**Keywords:** Cardioneuroablation, Ganglionated plexus, Reflex syncope, Catheter ablation

## Abstract

**Background:**

Radiofrequency ablation of the superior paraseptal ganglionated plexus attenuates vagal modulation of the sinus node (SAN). Ablation from the right-atrium aspect can achieve a sufficient effect. In a randomized study, we investigated the ablation effects of an anatomically prespecified right atrial linear lesion.

**Methods:**

Twenty patients (age 48 ± 13 years, 70% male) with recurrent reflex cardioinhibitory syncope or symptomatic sinus bradycardia underwent cardioneuroablation. A linear lesion consisting of six equidistantly spaced radiofrequency applications (30 W, 30 s, 20 mL/min) fully covering the posteroseptal quadrant of the superior vena cava (SVC) ostium. Patients were randomly assigned (1:1) to mutually opposite directions of lesion creation: posterior-to-septal (Group 1) or septal-to-posterior (Group 2). Sinus rate and SAN responses to extracardiac vagus nerve stimulation (ECVS) were recorded at baseline and after each radiofrequency energy delivery.

**Results:**

As expected, the complete lesion set in the total cohort significantly increased sinus rate (from 60 ± 10 to 80 ± 14 bpm; +35%; *P* < 0.0001) and reduced ECVS-induced sinus arrest (from 8.3 ± 1.7 to 2.2 ± 1.9 s; -74%; *P* < 0.0001). The most notable difference between the study groups was observed after performing the initial three lesions. Group 1 showed greater sinus rate acceleration (from 58 ± 9 to 80 ± 16 bpm; +39%; *P* < 0.001) than Group 2 (from 62 ± 10 to 72 ± 8 bpm; +18%; *P* < 0.01); *P* < 0.05 for between-group difference, and a more pronounced reduction in ECVS-induced sinus arrest (from 7.8 ± 1.4 to 1.4 ± 0.6 s; -82%; *P* < 0.0001) compared to Group 2 (from 8.9 ± 1.9 to 5.7 ± 3.0 s; -39%; *P* < 0.001); *P* < 0.0001 for between-group difference.

**Conclusions:**

Ablation of the posteroseptal quadrant of the SVC ostium significantly modified the vagal input to the sinoatrial node. Lesions targeting the posterior area produced more potent effects than those targeting the septal area.

**Graphical Abstract:**

**Figure:**
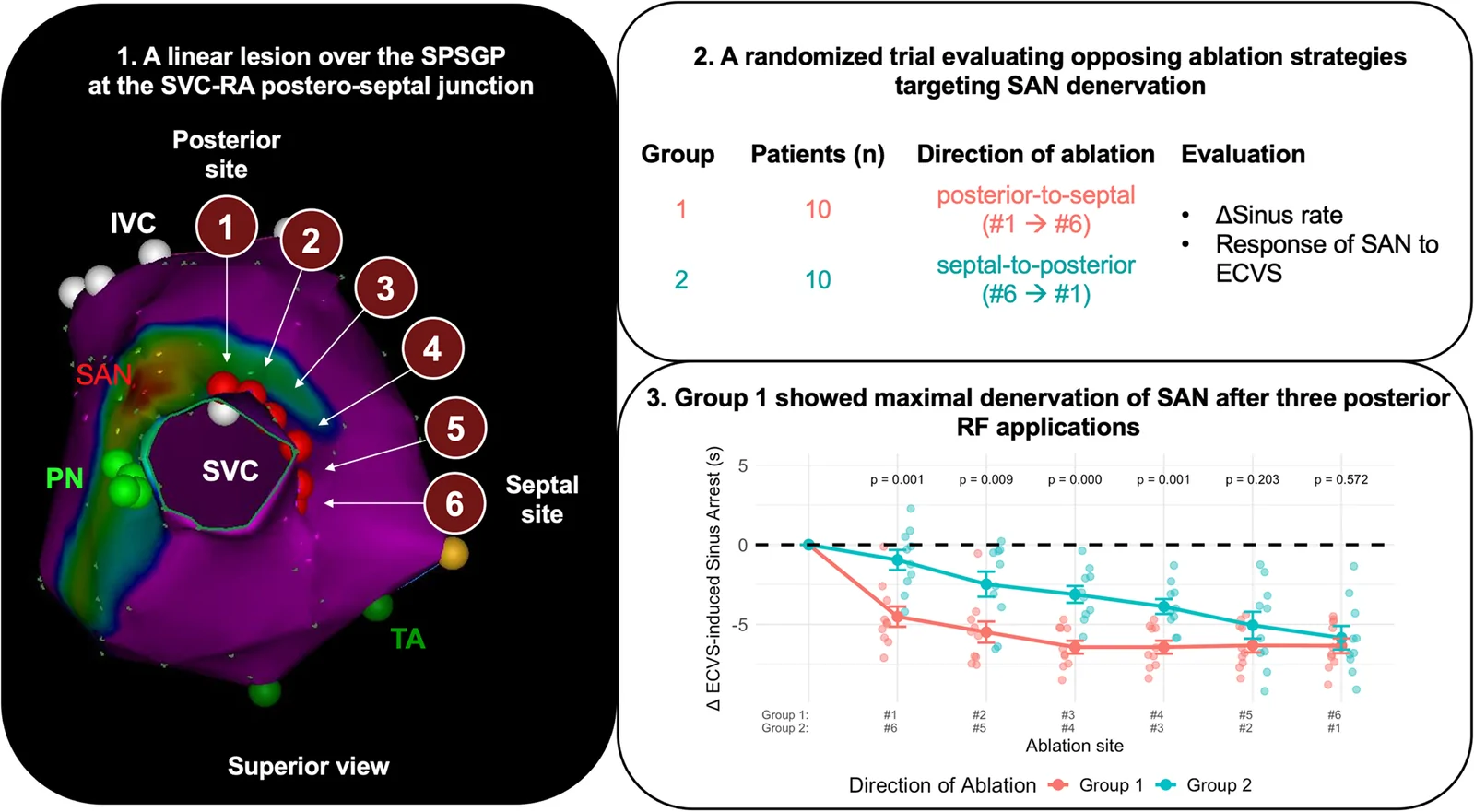

## Introduction

The superior paraseptal ganglionated plexus (SPSGP) is the primary target during cardioneuroablation (CNA) in patients with recurrent, treatment-resistant cardioinhibitory reflex syncope. This ganglionated plexus is located in an anatomical region bordered by the posteroseptal right atrium (RA) close to the junction with the superior vena cava (SVC), the left atrium adjacent to the anterior antrum of the right superior pulmonary vein, and the ascending aorta. The SPSGP contains a dense concentration of parasympathetic neurons that directly control the sinoatrial node (SAN). This is particularly important because sinus arrest due to exaggerated vagal activity is a leading factor in the development of reflex syncope in most of these patients [1–4]. 

CNA targeting the SPSGP offers an effective alternative to pharmacologic therapy or permanent pacemaker implantation. Radiofrequency ablation (RF) of SPSGP leads to suppression of parasympathetic modulation of the SAN with variable magnitude but reasonably sustained, resulting in a significant reduction in syncope burden [5–7].

Long-term outcomes from SPSGP-targeted CNA have been favorable. Studies consistently report freedom from syncope or bradyarrhythmic episodes. The procedure is well tolerated, with a low complication rate [6–9]. These features make SPSGP ablation particularly attractive in younger patients, where lifelong device therapy raises concerns about quality of life, durability, and safety [10, 11].

While various methods for localizing ganglionated plexi have been proposed – such as intracardiac high-frequency stimulation [12], evaluation of intracardiac electrograms [3], and computed tomography [13, 17] - empirical, anatomically-guided ablation remains the most commonly used approach [14]. 

Anatomical ablation of SPSGP from the aspect of the RA can produce reasonable results [14–17]. However, the optimal lesion set, which should be minimal in extent yet maximal in effect, has not been clearly established. Therefore, we conducted a randomized trial to investigate the burn-by-burn effects of ablation within a prespecified linear lesion from the RA endocardial aspect on the vagal modulation of the SAN.

## Methods

### Patients

The study enrolled patients referred to CNA. The inclusion criteria were recurrent reflex cardioinhibitory syncope or symptomatic functional sinus bradycardia documented by at least one of the following methods: a head-up tilt test, Holter ECG, implantable loop recorder, or vital function monitor. Eligible patients were over 18 years old, had no structural heart disease, and demonstrated a physiological response to the atropine test. This test involved intravenous administration of 2 mg of atropine (or 0.04 mg/kg in subjects weighing < 50 kg) with ECG recording before and 5 min after the bolus injection. The study received the institutional review board approval, and all patients provided written informed consent before the procedure.

### Preparation and technology

The procedure was conducted under general anesthesia. After obtaining venous access from both groins, a steerable decapolar catheter (Inquiry 6 Fr / 110 cm, St Jude Medical, USA) was placed in the coronary sinus for electrical reference. A 3.5-mm irrigated-tip Navistar Thermocool catheter (Biosense Webster, USA) was inserted via the Swartz SR0 introducer (Abbott, USA). A three-dimensional electroanatomic mapping and navigation system (CARTO-3, Biosense Webster) was utilized, and the procedure was facilitated by intracardiac echocardiography (ICE; Accunav, Siemens, USA).

### Extracardiac vagus nerve stimulation (ECVS)

The other steerable decapolar catheter (Inquiry 6 Fr / 110 cm, St Jude Medical) was inserted through the long sheath (Arrow-Flex 7 Fr / 80 cm, Teleflex, USA) to the right internal jugular vein. It was advanced almost to the cranial base to the proximity of the jugular foramen under fluoroscopy guidance and deflected in the posteromedial direction. ECVS was performed by the bipolar stimulation between ring electrodes 1 and 3 with a frequency of 50 Hz, a pulse width of 0.05 ms, an output of 1 V / kg body weight (max. 70 V), and a duration of 5 s, using a dedicated Extracardiac Autonomic Nerve Stimulator Pachon (Sao Paulo, Brazil) [18]. Micromanipulation of the catheter was used to maximize the response of SAN to ECVS. Once a reasonable magnitude of SAN response was obtained, a reference fluoroscopic image was stored and used to verify and adjust (in case of dislodgement) the catheter position before each subsequent ECVS testing.

### Cardioneuroablation

**Fig. 1:**
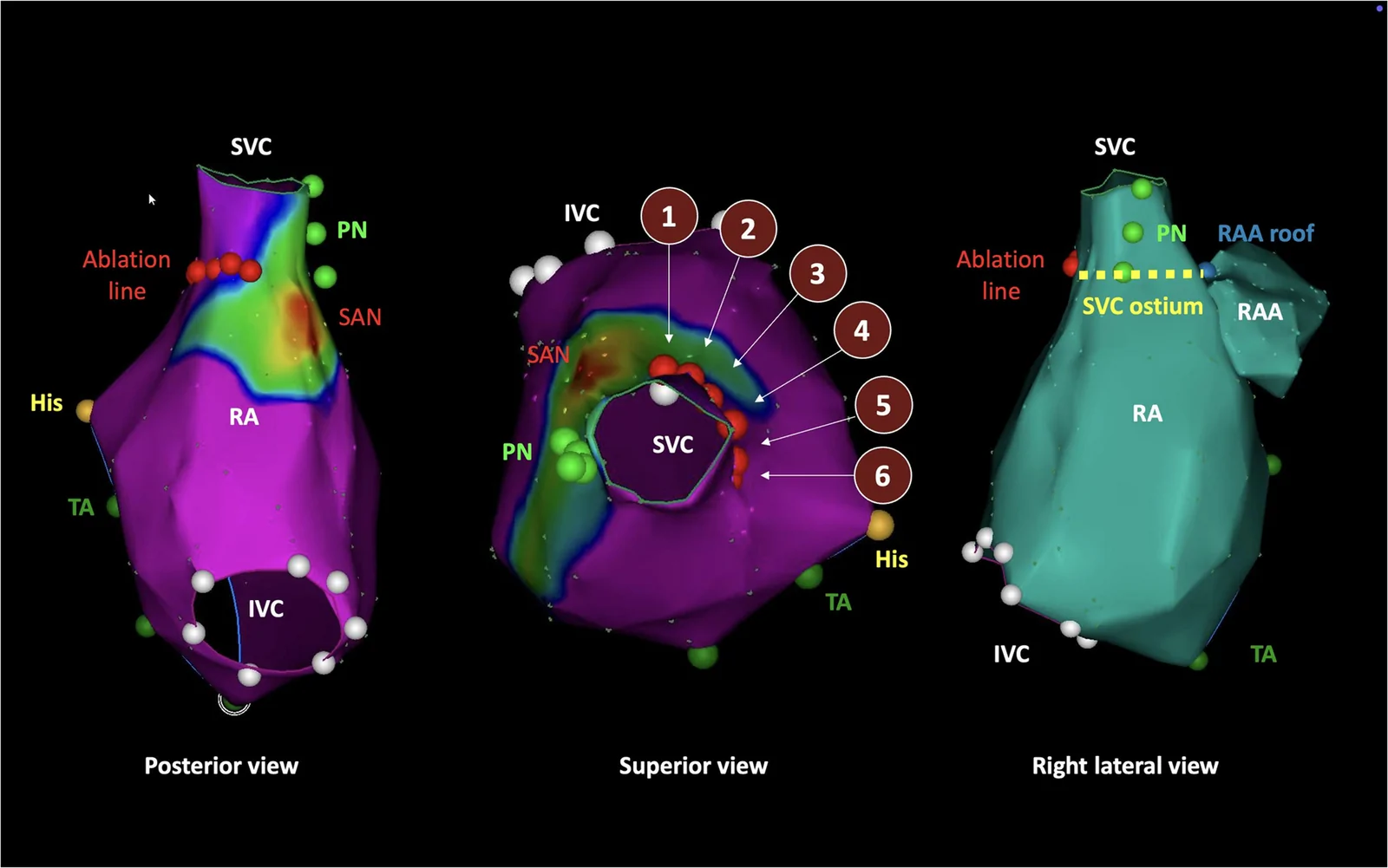
Study lesion set – Electroanatomic map of the right atrium with color-coded sinus activation and the semicircular ablation line covering the posteroseptal quadrant of the SVC ostium. Ablation sites are numbered from # 1 (posterior) to # 6 (septal) positions.IVC – inferior vena cava, PN – phrenic nerve, RAA – right atrial appendage, SVC – superior vena cava, TA – tricuspid annulus, SAN – sinus node

The RA was anatomically mapped, tagging sites with phrenic nerve capture (stimulation output of 15–20 mA). The SAN position was identified by activation mapping in sinus rhythm. The SVC ostium was tagged at the most proximal level, where the theoretical circular lesion could be performed in case of SVC isolation, i.e., defined by the ridge between the SVC and the RA appendage. For the study, a linear ablation lesion was designed in the posteroseptal quadrant of the SVC ostium, extending from the most posterior to the most septal site. This linear lesion comprised six equidistantly distributed ablation spots. In each spot, the RF energy was delivered for 30 s at an output of 30 W, with an irrigation rate of 20 mL/min (Fig. 1).

Patients were randomly assigned (1:1) to mutually opposite directions of ablation: posterior-to-septal (Group 1) or septal-to-posterior (Group 2), using simple randomization, ensuring each patient had an equal chance of being allocated to either group. Sinus rate and the SAN response to ECVS were outcome measures recorded at baseline and after each RF energy delivery. ECVS-induced sinus pause was measured as the longest interval between two P-waves, even in the presence of atrioventricular (AV) block. In addition, ECVS responses were quantified categorically. The prolongation of the sinus cycle length by less than 50% was arbitrarily considered a sufficient attenuation of the vagal innervation of SAN.

Once the study ablation was completed, the CNA procedure continued with a transseptal puncture, mapping of the left atrium and coronary sinus, and further ablation to finish our standard lesion set. This included mandatory biatrial SPSGP ablation, biatrial inferior paraseptal ganglionated plexus (GP) ablation, which always comprised ablation via the proximal coronary sinus, and optional left superior GP ablation. The endpoint of the procedure was the loss or significant attenuation of responsiveness of both SAN and AV node to ECVS.

### Statistical analysis

Statistical analysis was performed using R software version 4.4.2 (http://www.R-project.org, R Foundation for Statistical Computing, Vienna, Austria). Continuous data are presented as mean ± standard deviation. Categorical variables are presented as counts (percentages).

For the study lesions, the response of SAN to ECVS was quantified as the maximum induced P-P interval, or as its absolute or relative difference from baseline. The outcome measures were compared (i) between baseline and post-ablation time points using a t-test for dependent samples or Wilcoxon signed-rank test (as appropriate) and (ii) between groups using a t-test for independent samples or Mann-Whitney U test (as appropriate). A P-value < 0.05 was considered statistically significant. As this was a pilot study, a formal sample size calculation was not applicable.

For global CNA effects, the ratio of the maximum P-P interval to the current SR cycle length (PPratio) was used. This index is more sensitive for detecting residual vagal responses at advanced stages of the procedure, when the sinus rhythm is already accelerated, and absolute pause duration becomes less informative. A PPratio < 1.5 was arbitrarily chosen to indicate a marked attenuation of vagal responses. Such a cut-off corresponds to ECVS-induced instant heart rate slowing to no more than 50–55 bpm, which could be considered clinically nonsignificant.

## Results

This study included 20 patients (10 + 10 in both groups) with symptomatic bradyarrhythmias, primarily due to SAN disorder (Table 1). The baseline sinus rate and ECVS-induced sinus arrest were comparable between study groups (*P* = 0.36 and *P* = 0.20, respectively).

**Table 1:** Baseline characteristics of the study population

	All patients (*n* = 20)	Group 1 (*n* = 10)	Group 2 (*n* = 10)	*P*-value
Age, years	48 ± 13	47 ± 16	51 ± 12	0.28
Male, *n* (%)	14 (70)	7 (70)	7 (70)	1
SAN disorder, *n* (%)	12 (60)	7 (70)	5 (50)	0.39
AVN disorder, *n* (%)	7 (35)	3 (30)	4 (40)	0.66
SAN and AVN disorder, *n* (%)	1 (5)	0 (0)	1(10)	0.34
Number of syncope per patient/year before CNA	4 ± 3	4 ± 3	4 ± 4	0.65
Max. pause documented before CNA, s	9.0 ± 4.8	9.0 ± 2.8	9.0 ± 7.8	0.99
Atropine test – baseline heart rate, bpm	66 ± 17	69 ± 16	63 ± 17	0.39
Atropine test – post-atropine change, bpm	106 ± 20	113 ± 22	98 ± 14	0.09
Post-atropine relative change, %	65 ± 31	67 ± 32	62 ± 31	0.76

*AVN  *atrioventricular node, *SAN *sinoatrial node Data are provided as means ± standard deviations or counts (percentages)

### Overall study ablation effects

Study protocol ablations resulted in a significant sinus rate acceleration (from 60 ± 10 to 80 ± 14 bpm; +35%; *P* < 0.0001) and a reduction in ECVS-induced sinus arrest (from 8.3 ± 1.7 to 2.2 ± 1.9 s; -74%; *P* < 0.0001).

### Comparison of groups – overall study ablation effects

**Fig. 2:**
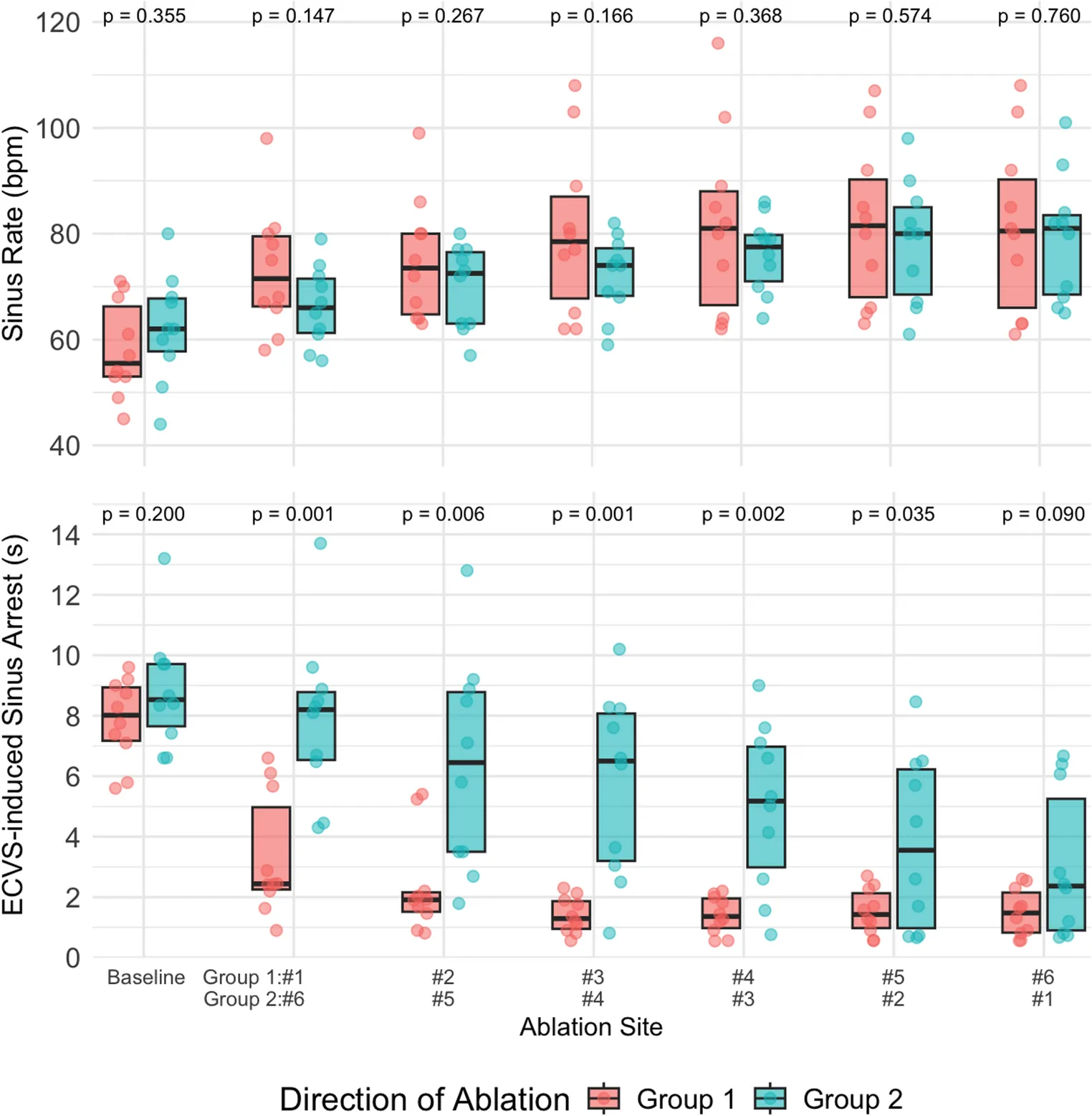
Progressive acceleration of sinus rate and attenuation of ECVS-induced sinus arrest (absolute values). Boxes represent the median and interquartile range. *P*-values are for between-group differences

**Fig. 3:**
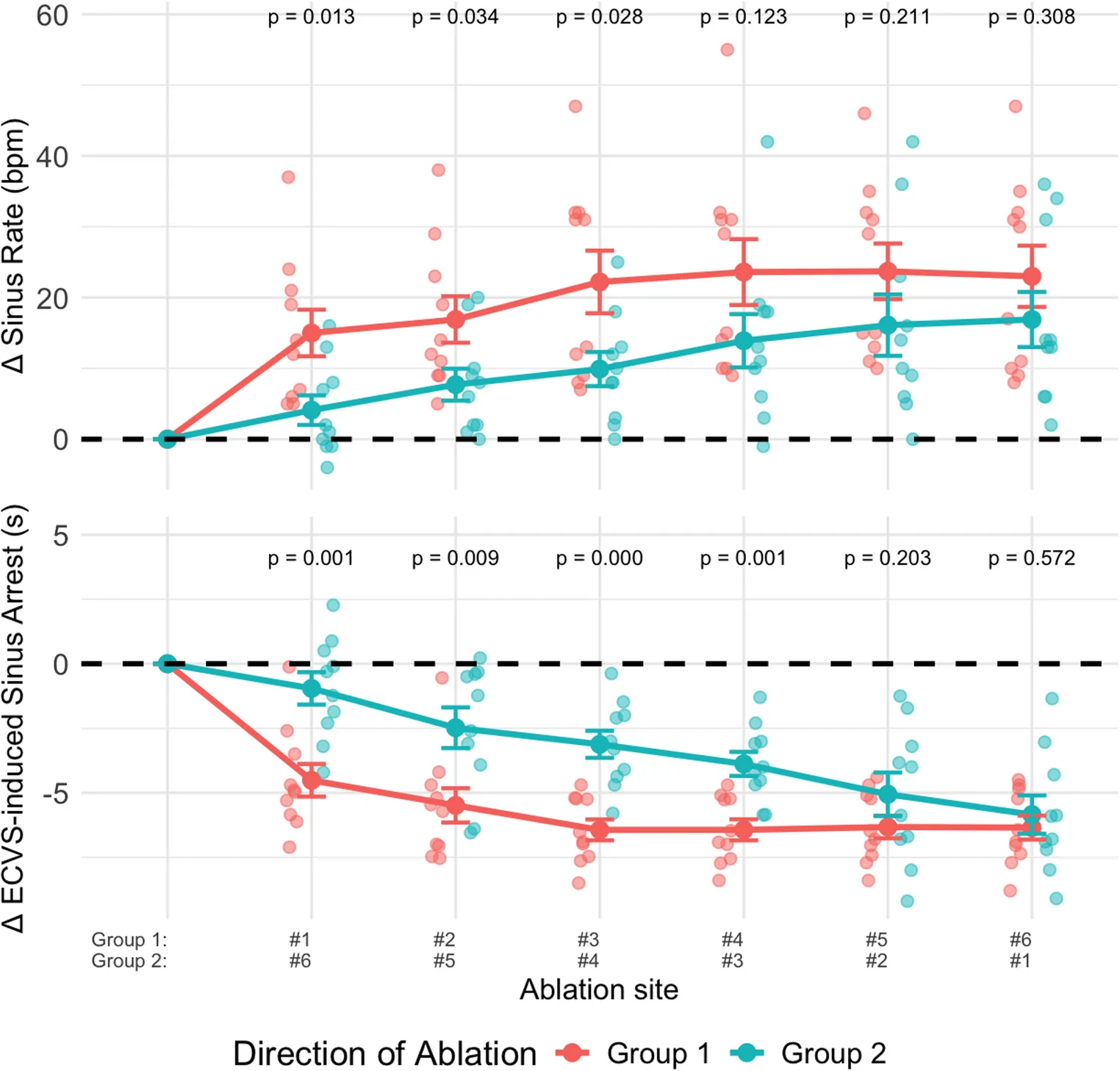
Progressive acceleration of sinus rate and attenuation of ECVS-induced sinus arrest (differences relative to baseline).Points with whiskers represent the mean with standard error. *P*-values are for between-group differences

A significant increase in sinus rate was observed in both study groups. This acceleration was numerically more prominent in Group 1 (from 58 ± 9 to 81 ± 16 bpm; +40%; *P* < 0.001) vs. Group 2 (from 62 ± 10 to 79 ± 12 bpm; +29%; *P* < 0.01). Additionally, there was a marked reduction in duration of ECVS-induced sinus arrest in Group 1 (from 7.8 ± 1.4 to 1.5 ± 0.8 s; -81%; *P* < 0.0001) vs. Group 2 (from 8.9 ± 1.9 s to 3.0 ± 2.4 s; -67%; *P* < 0.0001). However, the between-group differences were not statistically significant for the sinus rate and the duration of the ECVS-induced sinus arrest when comparing final absolute values (*P* = 0.76 and *P* = 0.09, respectively; see Fig. 2) or absolute difference from baseline (*P* = 0.31 and *P* = 0.57, respectively; see Fig. 3) or relative difference from baseline (*P* = 0.33 and *P* = 0.13, respectively). Categorically, sufficient attenuation of vagal innervation of SAN was achieved in 4 of 10 patients in both study groups.

### Comparison of groups – temporal development of study ablation effects

The temporal development of outcome measures varied between study groups, as illustrated in Figs. 2 and 3. The most significant differences were observed after performing the initial three RF applications. At this time point, Group 1 showed greater sinus rate acceleration (from 58 ± 9 to 80 ± 16 bpm; +39%; *P* < 0.001) than Group 2 (from 62 ± 10 to 72 ± 8 bpm; +18%; *P* < 0.01). Additionally, there was a marked reduction in ECVS-induced sinus arrest in Group 1 (7.8 ± 1.4 to 1.4 ± 0.6 s; -82%; *P* < 0.0001) compared to Group 2 (from 8.9 ± 1.9 to 5.7 ± 3.0 s; -39%; *P* < 0.001). The between-group differences were mostly significant for the sinus rate and the duration of the ECVS-induced sinus arrest when comparing final absolute values (*P* = 0.16 and *P* < 0.001, respectively; see Fig. 2) or absolute difference from baseline (*P* < 0.05 and *P* < 0.0001, respectively; see Fig. 3) or relative difference from baseline (*P* < 0.05 and *P* < 0.0001, respectively). ECVS after study ablation provided the PPratio of 1.9 ± 0.9 in Group 1 and 3.7 ± 2.7 in Group 2 (*P* = 0.06). In both groups, significant attenuation of vagal responses (PPratio < 1.5) was achieved in only 4/10 (40%) patients.

### Final ablation effects

After performing additional left atrial SPSGP ablation and biatrial inferior paraseptal GP ablation, there were no significant differences in sinus rate between Groups 1 and 2, with rates of 80 ± 14 bpm and 79 ± 9 bpm, respectively (*P* = 0.79). The PPratio decreased to 1.1 ± 0.2 in Group 1 and 1.7 ± 1.9 in Group 2 (*P* = 0.30) during the final ECVS. Significant attenuation of vagal responses (PPratio < 1.5) was achieved in 10/10 (100%) patients in Group 1 and 8/10 (80%) patients in Group 2.

### Safety, complications, and follow-up

No patient needed modification of the study lesion set due to co-localization with the SAN or the phrenic nerve. No complications were observed during the study. Over a follow-up period of 42 ± 23 months, symptomatic bradyarrhythmia recurred in 3 patients. One patient in Group 1 was successfully reablated. In Group 2, one patient had a pacemaker directly implanted, and another required pacemaker implantation after a failed repeat CNA.

## Discussion

The study showed that the anatomically defined set of ablation lesions in the posteroseptal quadrant of the SVC ostium had a significant though incomplete effect on the vagal innervation of the SAN. The key finding of the study is that posterior lesions were more effective than septal lesions, as evidenced by burn-by-burn assessment of sinus rate acceleration and the reduction of ECVS-induced sinus arrest.

### Design of RA ablation

The effectiveness of RA ablation is not surprising, as previous studies have demonstrated reasonable outcomes of CNA using a limited, unifocal RA ablation [6, 7]. However, these studies used a CT-guided approach, and their description of the targeted area – posteroseptal junction between the RA and SVC, adjacent to the mid and caudal regions of the right superior pulmonary vein antrum – was relatively vague. In contrast, our lesion set was precisely defined based on RA anatomy using 3D endocardial mapping, without the assistance of CT imaging. The SVC ostium was identified using ICE, but this can also be achieved simply by catheter manipulation at the SVC/RAA ridge.

### Rationale for study lesion set

There are several reasons why we chose this particular lesion set. First, the posteroseptal quadrant of the SVC ostium, along with the superoseptal left atrium and ascending aorta, defines a triangular space that contains most of the neural tissue belonging to SPSGP. Second, we aimed to avoid ablating deeper inside the SVC to minimize the risk of developing SVC stenosis. It is also known that the atrial wall thickness decreases when one navigates from the RA body to the SVC. Therefore, the SVC ostium is considered the most appropriate location for ablation, as effective targeting of epicardial neural tissue requires at least a transmural lesion. Third, we believe that creating a semicircular linear ablation lesion perpendicular to the long SVC axis transects the greatest proportion of vagal axons that approach the heart, mostly in a craniocaudal direction. Finally, we selected the posteroseptal quadrant of the SVC ostium for safety reasons. Such a lesion should not coincide with the earliest endocardial activation sites during sinus rhythm and/or sites with phrenic nerve capture. Indeed, we did not have to modify this lesion and did not observe damage to the SAN or phrenic nerve palsy in any patient.

### Ablation outcome measures

Unlike previous studies, our study design incorporated “bidirectional” ablation and assessed technical outcome measures using ECVS after each RF application. This approach allowed us to identify the most effective ablation sites. As anticipated, the duration of ECVS-induced sinus arrest better discriminated between study groups in terms of ablation effects than sinus rate. This finding is plausible because the ECVS-based measure provides a more sensitive and specific surrogate of current SAN vagal modulation. This is also consistent with our previous study, which showed poor agreement between changes in sinus rate and attenuation of the SAN response to ECVS during stepwise CNA [19]. In general, the utility of ECVS in mechanistic studies is also supported by better clinical outcomes after ECVS-guided CNA procedures [20].

### Efficacy of study ablation

Compared to septal ablation sites, posterior ablation sites were found to be more effective in reducing vagal modulation of the SAN. As a result, patients in Group 1 experienced nearly the maximum effect early in the study ablation (after the creation of posterior lesions), and additional septal ablations did not further enhance the total effect. This implies that targeting only the posterior part of the study lesion set can be sufficient to achieve a reasonable modification of SPSGP. It is important to note, however, that ablation of septal sites is not ineffective, as in patients from Group 2, the initial septal lesions produced a significant (approximately half of the total) effect.

The results indicate that a strategically placed and limited lesion set can be as effective as a large cluster of lesions. By identifying and targeting the most impactful ablation sites – referred to as “sweet spots” – early in the procedure, unnecessary lesions can be avoided. This approach helps limit myocardial scarring, minimize potential complications from collateral damage, and reduce procedural time while still achieving significant attenuation of vagal modulation of the SAN.

The overall efficacy of the study ablation was rather modest, as the lesion was intentionally designed to prevent ablative oversaturation and, consequently, to assess the differential effect of individual RF applications. This may contrast with a strategy of limited but much deeper RA lesions [17] or extensive RA posteroseptal ablation [8], which can achieve much stronger effects, although not formally tested by ECVS.

### CNA beyond the study ablation

After completing the study ablation, we systematically continued with biatrial CNA targeting the innervation of both the SAN and the AVN. This is our standard strategy in order to (1) enhance the effect of RA ablations, which is in line with earlier research by Piotrowski et al. [21] and Francia et al. [22], and (2) prevent the recurrence of functional AV block in patients who initially manifested as a sinus nodal disorder, and vice versa. Such a strategy is justifiable by evidence that individual ganglionated plexi exert selective yet overlapping influences on SAN and AV node function and that incomplete denervation may unmask latent abnormalities, particularly involving the AV node [23, 24].

### Limitations

This study has several limitations. First, it focused on the acute changes in vagal modulation of the SAN, which may not reflect the long-term effects. Second, the study lesion may have primarily damaged preganglionic axons entering the SPSGP, which may have a high potential for regeneration. Third, the study did not intentionally investigate the impact of study ablations on the AV node, which is well known. However, such an investigation would have required doubling the number of ECVS tests, as these had to be repeated during atrial pacing. Fourth, the small sample size reflects the exploratory design of this mechanistic study and limits statistical power to detect differences beyond “posterior vs. septal” categories, i.e., to identify the RA ablation sweet spot more precisely. Fifth, the lack of standardized ablation parameters due to the absence of contact force sensing (and ablation index feedback) might affect the uniformity and efficacy of the ablations. Sixth, an additional CNA on top of study ablations effectively prevented inter-group comparisons of clinical outcomes. Finally, the investigated lesion set performed from the aspect of the RA was only one of numerous possible configurations, some of which might have comparable or even higher efficacy.

## Conclusions

Ablation of the posteroseptal quadrant of the SVC ostium significantly modifies vagal input to the SAN. Posterior ablation sites exerted the strongest immediate effect on vagal modulation of the SAN, and can be considered a sufficient target for SPSGP denervation from the endocardial aspect of the RA. Further studies are needed to determine whether such a limited lesion set can provide consistent and durable clinical effects.

## Data Availability

The data that support the findings of this study are not openly available due to reasons of sensitivity and are available from the corresponding author upon reasonable request.

## Abbreviations

AV: Atrioventricular
CNA: Cardioneuroablation
ECVS: Extracardiac Vagus Nerve Stimulation
GP: Ganglionated Plexus
PPratio: Ratio Between the Maximum ECVS–Induced P–P Interval and the Current SR Cycle Length
RA: Right Atrium
RF: Radiofrequency
SAN: Sinoatrial Node
SPSGP: Superior Paraseptal Ganglionated Plexus
SVC: Superior Vena Cava
